# Administration of FOLFIRINOX for Advanced Pancreatic Cancer: Physician Practice Patterns During Early Use

**DOI:** 10.3390/curroncol32030128

**Published:** 2025-02-25

**Authors:** Joanna Gotfrit, Horia Marginean, Yoo-Joung Ko, Akmal Ghafoor, Petr Kavan, Haji Chalchal, Shahid Ahmed, Karen Mulder, Patricia Tang, Rachel Goodwin

**Affiliations:** 1 The Ottawa Hospital Cancer Centre (TOHCC), Ottawa, ON K1H 8L6, Canada; 2 Unity Health, Toronto, ON M5B 1W8, Canada; 3 Windsor Regional Hospital (WRH), Windsor, ON N8W 2X3, Canada; 4 Jewish General Hospital (JGH), Montréal, QC H3T 1E2, Canada; 5 Allan Blair Cancer Centre (ABCC), Regina, SK S4T 7T1, Canada; 6 Saskatoon Cancer Center (SCC), Saskatoon, SK S7N 4H4, Canada; 7 Cross Cancer Institute (CCI), Edmonton, AB T6G 1Z2, Canada; 8 Arthur J. E. Child Comprehensive Cancer Centre (ACCC), Calgary, AB T2N 5G2, Canada

**Keywords:** FOLFIRINOX, advanced pancreatic cancer, clinical practice, practice patterns, prescribing

## Abstract

Advanced pancreatic cancer results in high morbidity and mortality. The standard of care treatment in the advanced setting changed in 2011 with the introduction of FOLFIRINOX (FFX) chemotherapy. However, it was highly toxic with significant risk of complications. We assessed the practice patterns of medical oncologists across Canada. Methods: We performed a retrospective study of consecutive patients with advanced pancreatic cancer treated with FFX at eight Canadian cancer centers. Demographic, treatment, and outcome data were collected and analyzed. Results: The median age of the patients was 61 (range 24–80), 43% were female, 96% had an ECOG PS of 0 or 1, and 50% had three or more metastatic sites. The median follow-up time was 20.8 months (95%CI 18.6–24.9). Physicians started FFX at the standard dose 31% of the time. Physicians prescribed GCSF for primary prophylaxis most when giving standard-dose FFX (30% of the time) in comparison to reduced dose with or without the 5-FU bolus. Dose reductions occurred in 78.1% of patients, while dose delay occurred in 65.2% of patients. Conclusions: Medical oncologists in Canada historically prescribed FFX to patients with advanced pancreatic cancer in a fashion that was not uniform, prior to the emergence of evidence for upfront dose reductions.

## 1. Introduction

Pancreatic cancer is the twelfth most commonly diagnosed cancer in Canada and the fourth leading cause of cancer-related death. Only about half of patients live beyond 4 months from the time of diagnosis. The 5-year net survival rate is 7%, giving it the worst prognosis of all cancers assessed by the Canadian Cancer Society. The high mortality rate is attributable to the fact that more than 60% of pancreatic cancers are diagnosed at an advanced stage, reducing the chance of successfully treating it with surgery [1]. It is also relatively resistant to treatment with chemotherapy [2].

In modern practice, there are two main evidence-based first-line chemotherapy treatments for metastatic pancreas cancer. These include FOLFIRINOX (FFX) [3] or Gemcitabine plus nab-Paclitaxel [4]. These regimens demonstrate overall survival of less than 1 year in clinical trials. Radiation may also be used primarily for symptom control and quality of life. Molecular testing is highly encouraged to identify potentially actionable mutations. Clinical trials should be sought when possible [5]. Pancreas cancer remains a relatively treatment-resistant and poor-prognosis cancer.

Prior to 2011, there were few chemotherapeutic options for patients with advanced pancreatic cancer, and available options had limited efficacy. The main available option was single agent Gemcitabine, proven in a 1997 clinical trial to have greater clinical benefit than single agent 5-fluorouracil and to have a median survival of 5.65 months [6]. The therapeutic landscape changed considerably with the introduction of FFX in the landmark ACCORD study by Conroy et al. [3] in 2011, where the triplet chemotherapy combination of 5-fluorouracil (bolus and infusional), irinotecan, and oxaliplatin extended the median overall survival (mOS) of patients with metastatic pancreatic cancer (mPC) from approximately 7 months to 11 months, making it a new standard of care. The ACCORD study included only patients with metastatic disease; however, subsequent research has assessed FFX as a first-line regimen for patients with locally advanced disease (LAPC; localized but unresectable), and FFX is now recommended as a first-line standard of care option in this setting as well [7,8,9].

While the incremental survival benefit of FFX is impressive, patients treated with this regimen experience high rates of grade 3 and 4 adverse events, including neutropenia (46%), fatigue (24%), vomiting (15%), diarrhea (13%), thrombocytopenia (9%), neuropathy (9%), and febrile neutropenia (5%) [3]. In a Canadian real-world analysis, more than half of patients on FFX were hospitalized (mostly due to sepsis) and 7% of patients experienced treatment-related death [10]. As such, modifications to dose and schedule have been proposed and implemented at some institutions and by some individuals to improve the safety profile, particularly with regards to myelosuppression. Some advocate for overall dose reductions, elimination of the 5-FU bolus, and/or use of prophylactic granulocyte colony stimulating factor (GCSF) [11].

While cross-trial comparisons are imperfect, toxicities of FFX [3] tend to be greater than other first-line options such as Gemcitabine plus nab-Paclitaxel [4]. For example, the grade 3 or 4 neutropenia rate for FFX is 47.5% and for Gemcitabine plus nab-Paclitaxel is 38%. The febrile neutropenia rates are 5.4% and 3%, respectively. The grade 3 or 4 diarrhea rate for FFX is 12.7% and for Gemcitabine plus nab-Paclitaxel is 6%.

While dose reductions and drug omissions may improve safety, as shown in ref. [11], previous research in a variety of malignancies has demonstrated compromised survival associated with regimen alterations, as measured by changes in dose intensity (DI) [12] and relative dose intensity (RDI) [13]. It has been shown that patients with epithelial ovarian cancer receiving an RDI of ≤0% had significantly poorer survival than those receiving an RDI of 91–100% [14,15]. Similar results have been demonstrated in a multitude of other malignancies in both the adjuvant and metastatic settings [16,17,18].

The purpose of the present study was to describe the practice patterns of medical oncologists in Canada with regard to FFX administration in the first-line setting of metastatic pancreatic cancer and locally advanced pancreatic cancer at the time when FFX was newly available.

## 2. Materials and Methods

### 2.1. Study Population

With ethics approval from all participating institutions (REB approval numbers in brackets), we performed a retrospective, multi-institutional, observational, and longitudinal study of consecutive patients with newly diagnosed advanced pancreatic cancer treated with FFX at eight Canadian cancer centers: The Jewish General Hospital in Montreal, Quebec (CR 12-60); The Ottawa Hospital Cancer Centre in Ottawa, Ontario (2011871-01H); The Windsor Regional Hospital in Windsor, Ontario (CC-13-133); Sunnybrook Odette Cancer Centre in Toronto, Ontario (157-2013); The Saskatoon Cancer Centre in Saskatoon, Saskatchewan (14-178 SCC); The Allan Blair Cancer Centre in Regina, Saskatchewan (14-178 ABCC); the Tom Baker Cancer Centre in Calgary, Alberta (26219); and the Cross Cancer Institute in Edmonton, Alberta (26219). Patients were enrolled between 17 December 2012 and 7 January 2015.

Patients were included if they had previously untreated unresectable pancreatic cancer, either mPC or LAPC. Patients were excluded if they had received previous treatment for mPC or LAPC prior to FFX. Patients who received previous systemic therapy before FFX were included if the systemic therapy was due to delay receiving FFX and did not exceed 2 cycles. Previous adjuvant therapy was allowed.

The patients’ clinical data were collected by retrospective medical chart review and included baseline demographics (age, sex, Eastern Cooperative Oncology Group performance status (ECOG PS), body mass index (BMI, tumor characteristics (anatomic location of primary tumor, tumor stage based on AJCC 6th edition, tumor size, histology, grade, extent of disease), treatment variables (modification of starting dose or subsequent cycles, G-CSF use), and toxicities leading to treatment discontinuation (hematologic and non-hematologic).

The ACCORD trial by Conroy et al. [3] established FFX as a standard of care in the treatment of patients with metastatic pancreatic cancer. In our study, patients were grouped into ACCORD positive and ACCORD negative subgroups based on their characteristics. Patients classified as ACCORD positive were those who met the inclusion criteria for the ACCORD trial: age 18–75 with metastatic pancreatic adenocarcinoma, ECOG 0–1, adequate blood counts, adequate liver function, adequate kidney function, absence of previous radiotherapy for measurable lesions, absence of cerebral metastases, absence of another major cancer, absence of infection, absence of chronic diarrhea, absence of significant cardiac disease, and absence of pregnancy/breastfeeding. Patients that did not satisfy the inclusion criteria for the trial were classified as ACCORD negative: no adenocarcinoma, age greater than 75, previous systemic therapy before FFX, ECOG PS greater than 1, and abnormal laboratory values. Those who had LAPC were grouped in their own category, as the trial did not include patients with LAPC.

### 2.2. Chemotherapy Dosing

The FOLFIRINOX regimen as described by Conroy et al. in the ACCORD trial [3] was considered to be the standard regimen: oxaliplatin 85 mg/m^2^, leucovorin 400 mg/m^2^, irinotecan 180 mg/m^2^, fluorouracil bolus 400 mg/m^2^, and fluorouracil infusion 2400 mg/m^2^ over 46 h, with all drugs repeated every 14 days.

Dose reduction was defined as a reduction of at least 10% of the overall FFX dose. Dose intensity (DI) was defined as the proportion of total dose administered without consideration for dose delays. Relative dose intensity (RDI) was defined as the proportion of total dose administered considering dose delays. Intervals between chemotherapy administrations and drug dose based on true body weight were independently recorded to calculate DI [12] and RDI [13] as previously described. To summarize, DI was calculated by dividing the amount of drug delivered per interval over the amount planned. RDI was calculated by multiplying the DI by the ratio of the expected duration for delivery over the interval of drug delivery. The DI and RDI for the whole FFX regimen were calculated as the mean of the dose intensities of the individual drugs that compose the regimen.

### 2.3. Statistical Analysis

We separated patients into groups, based on (1) whether they fulfilled the ACCORD trial inclusion criteria (ACCORD positive) or not (ACCORD negative), and (2) FFX starting dose as used by the ACCORD trial: standard dose, reduced dose, or FFX without 5-FU bolus. Baseline characteristics and treatment outcomes were compared. Continuous variables were presented as median and range and categorical variables were presented as numbers and percentages. A *p*-value < 0.05 (two-tailed) was considered significant. Confidence intervals (CI) were provided at 95% CI. The median follow-up time was calculated using the Kaplan–Meier curve with the reversed status indicator proposed by Schemper and Smith [19]. Data analysis was performed using Stata version 16.

## 3. Results

A total of 224 patients treated in eight hospitals from 4 Canadian provinces were eligible for analysis.

### 3.1. Study Population Characteristics

In the overall cohort, the median age was 61 years (range 24–80), 43% were female, 96% had an ECOG PS of 0 or 1 at the time of FFX initiation, and 50% had three or more metastatic sites. The median follow-up was 20.8 months (95%CI 18.6–24.9). Baseline characteristics are summarized in Table 1.

Baseline demographics among patients who received standard- vs. reduced-dose FFX vs. FFX with the 5-FU bolus omitted were generally similar with the exception of the gender, ECOG PS, similarity with the ACCORD trial, and number of metastatic sites, as shown in Table 2. Baseline demographics among patients who were categorized as ACCORD positive vs. ACCORD negative vs. LAPC were generally similar except for age and ECOG PS, as shown in Table 2.

#### 3.1.1. FFX Administration Practice Patterns

Physicians started FFX at a standard dose less than half the time (31%). They started at a reduced dose 28% of the time and omitted the 5-FU bolus 40% of the time. Physicians started with more than a 30% dose reduction in 35% of patients. Median number of treatments was seven cycles (range 1–48) lasting a median duration of 8.4 months (range 0.5–35.9). The first dose reduction occurred at a median cycle of 8 (range 1–32) and the first dose delay occurred at a median cycle of 5 (range 1–22). Granulocyte colony stimulation factor (GCSF) was used as primary or secondary prophylaxis in 41% of patients. FFX administration by physicians is summarized in Table 3 and the relative dose intensity is shown in Table 4.

#### 3.1.2. Use of Granulocyte Colony Stimulating Factor

**Figure 1:**
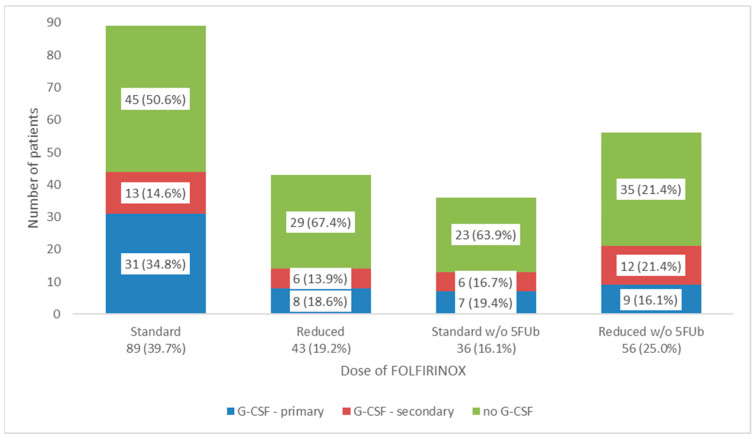
Use of primary and secondary GCSF by starting dose. Abbreviations: w/o, without; 5Fub, 5-fluorouracil bolus; GCSF, granulocyte colony stimulating factor.

Physicians prescribed GCSF for primary prophylaxis most when giving standard-dose FFX (30% of the time) in comparison with reduced dose with or without the 5-FU bolus. Patients who received standard-dose FFX were also most likely to receive secondary prophylaxis with GCSF (19% of the time), whereas patients receiving reduced-dose FFX with or without the 5-FU bolus were less likely to receive secondary prophylaxis with GCSF. See Figure 1 for a breakdown of patients receiving primary and secondary GCSF by starting dose category.

#### 3.1.3. Dose Adjustments and Treatment Discontinuation

Dose reductions occurred in 175 (78.1%) of patients, while dose delay occurred in 146 (65.2%) of patients. Early FFX discontinuation (before 12 cycles) occurred in 26 (11.6%) of patients, 12 of whom (46.2%) were started on the standard dose.

**Figure 2:**
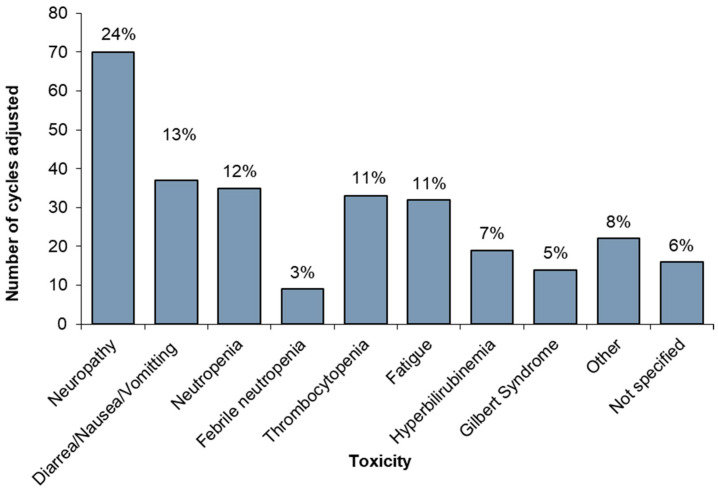
Dose adjustments due to toxicity.

Of 2019 cycles of FFX delivered, 677 (34%) cycles were delivered with at least 1 drug adjusted (delay, reduction, or discontinuation). Of these, 288 cycles (14% of all cycles given to the whole cohort) were delivered with a drug adjustment due to toxicity. The reason for drug adjustment for the remainder of cycles was unclear from the charts. Among the total number of cycles adjusted for toxicity (n = 288), the most common reason for dose adjustment was neuropathy (24%) followed by diarrhea/nausea/vomiting (13%), and neutropenia (12%). Figure 2 demonstrates the number and proportion of cycles adjusted due to each toxicity.

**Figure 3:**
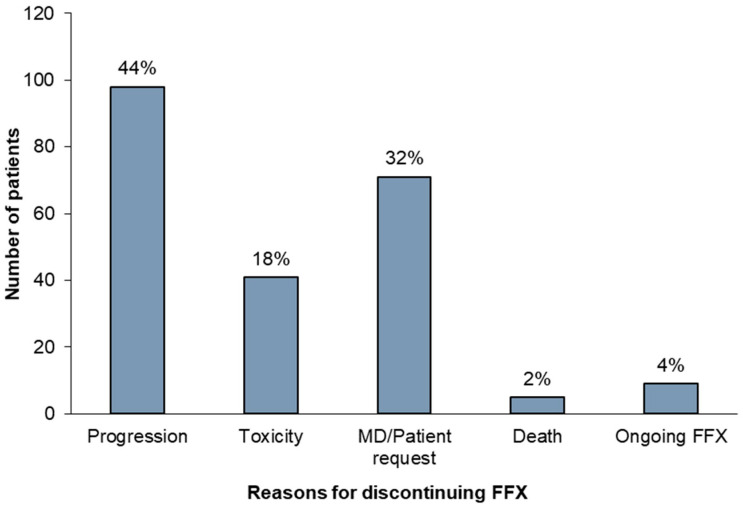
Reasons for FFX discontinuation. Abbreviations: MD, medical doctor; FFX, FOLFIRINOX.

At a median follow-up of 20.8 months, 215 patients had discontinued FFX (96% of overall cohort). The most common reason for FFX discontinuation was progression (43.7%), followed by MD/patient request without explicitly stated reason (31.7%), or toxicity (18.3%). See Figure 3 for reasons for discontinuation.

**Figure 4:**
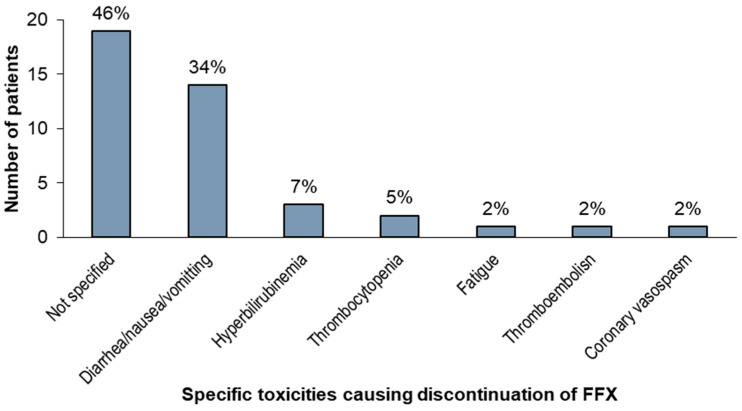
Toxicities leading to discontinuation of FFX among patients discontinuing treatment due to toxicity. Abbreviations: FFX, FOLFIRINOX.

Among patients who discontinued FFX due to toxicity (n = 41), the specific toxicity was not recorded in almost half (46%); however, 34% discontinued treatment due to diarrhea/nausea/vomiting, 7% due to hyperbilirubinemia, and 5% due to thrombocytopenia. See Figure 4 for the toxicities leading to discontinuation.

## 4. Discussion

When FFX became a new first-line standard of care chemotherapy regimen in advanced pancreatic cancer with the publication of the ACCORD trial by Conroy et al. in 2011 [3], there was concern that the regimen was excessively toxic based on high rates of grade 3 and 4 adverse events. As the uptake of FFX increased over time [20], some cancer centers and individual medical oncologists across Canada adjusted the starting dose, creating variability in FFX administration across the country. Given that this was a fairly novel regimen, centers developed their own cultures and practice patterns around its use, which helped the oncology community learn about best practices through experience. Similarly, at the time of FFX initiation, there were no formal guidelines on best practices for use, also predisposing to variable practices. We described the practice patterns of medical oncologists across Canada from 2012–2015 at the time FFX was newly used in this setting.

Within our cohort of patients at eight Canadian Cancer Centers across four Canadian provinces, which likely represents some of the first patients in Canada to receive FOLFIRINOX in the metastatic setting, many patients (37%) had metastatic disease but did not fulfill criteria set out in the ACCORD trial [3], indicating that these patients would not have been eligible for trial enrollment. This suggests that medical oncologists in Canada used more lenient selection criteria when determining eligibility for FFX in the real world, as is the case for other effective treatments in cancer [21]. For example, the ACCORD trial [3] included only patients < 76 years of age, while our cohort of treated patients reveals that 7% were 76 years of age and older. Age was not correlated with the starting dose of FFX, as the median age of patients starting full-dose FFX vs. FFX with dose reductions/omissions was similar (around 60 years). Patients receiving an upfront reduced dose of FFX tended to be female, have PS 1 (vs. 0), and have disease with a higher number of metastatic sites. This suggests the possibility that oncologists perceived female patients with worse performance status and/or a high burden of disease to be more frail at the outset of treatment, necessitating upfront regimen alterations.

We found that physicians started FFX at the standard dose (the dose used in the ACCORD trial [3]) less than half of the time. Most commonly, they started with a dose reduction in one or more drugs and/or omission of the 5-FU bolus. In about one-third of patients, the starting dose for the whole regimen was less than 70% of the standard dose. This suggests that medical oncologists in Canada were indeed concerned about the toxicity profile of FFX and made upfront adjustments to prevent toxicity. Despite the high rate of upfront dose reductions/omissions, of over 2000 total cycles of FFX delivered to the cohort, approximately one out of every seven cycles was delivered with at least one drug adjusted due to toxicity (most often neuropathy, diarrhea/nausea/vomiting, or neutropenia). Similarly, approximately one out of every five patients stopped the regimen due to toxicity. As such, the high rate of upfront regimen alterations did not eliminate the possibility of important side effects causing further dose adjustments and discontinuation of chemotherapy. That said, the toxicity seen in our cohort was substantially less than that seen in a real-world prospective Japanese cohort treated with standard-dose FFX, where 78% experienced grade 3 or 4 neutropenia and 22% experienced febrile neutropenia, resulting in dose reductions/delays in 89% of patients [22]. Toxicity was substantially reduced without compromising outcomes in a similar Asian cohort treated with modified dose FFX [23].

The previous literature in both the metastatic and adjuvant settings of various malignancies has demonstrated the reduced efficacy of dose-adjusted chemotherapy [14,15,16,17,18]. Within pancreatic cancer specifically, a retrospective analysis has shown that a cumulative RDI of >70% is necessary to maintain an optimal response rate, and >55% is needed to maintain an optimal disease control rate [24]. In our cohort, it seems that most patients received an RDI > 70%; however, a substantial proportion did not. This calls for the development of guidelines addressing appropriate starting dosage. The 2016 American Society of Clinical Oncology (ASCO) guidelines on treatment of metastatic pancreatic cancer suggested use of standard-dose FFX, but noted that omission of the 5-FU bolus and leucovorin could be considered early (including at the time of treatment initiation). The guideline went on to suggest dose reductions for irinotecan and/or oxaliplatin based on emerging toxicities [25]. The 2020 guideline update makes no further comment on the dosing of FOLFIRINOX in the metastatic setting [5].

More recently, another trial by Conroy et al. [26] demonstrated efficacy of FFX in the adjuvant setting of pancreatic cancer using a modified dosing regimen consisting of 5-FU 2400 mg/m^2^ infusion over 46 h (without a bolus), irinotecan 150 mg/m^2^, and oxaliplatin 85 mg/m^2^. Compared to standard-dose FFX, this modified FFX effectively equates to a lower RDI. A real-world retrospective review demonstrated similar efficacy of modified FFX (defined as 75% dose intensity) compared to standard-dose FFX in the setting of advanced pancreatic cancer, but with less toxicity [27]. This has been confirmed in a meta-analysis [28]. These data suggest that modified FFX may be a reasonable option in the setting of advanced disease as well. Similarly, there is some early evidence that patient-tailored dosing of FFX may provide better outcomes than classic FFX [29].

In our cohort, patients who received standard-dose FFX were more likely to be prescribed primary prophylaxis GCSF than those who received reduced-dose FFX or FFX without the 5-FU bolus. Interestingly, while the rate of neutropenia associated with standard-dose FFX is known to be high (46% in the ACCORD trial [3]), the rate of febrile neutropenia is known to be only 5% and does not meet the ASCO guidelines for routine use of primary GCSF prophylaxis (rate of febrile neutropenia > 20%) [30]. The 2016 and 2020 ASCO guidelines [5,25] on treatment of patients with advanced pancreatic cancer did not advocate for routine use of primary prophylaxis GCSF, suggesting drug modifications instead for blood counts precluding administration of chemotherapy. Despite the guidelines, the relatively frequent use of primary prophylaxis GCSF by medical oncologists in Canada may suggest that real-world experience with FFX leads to more infection-related toxicity than predicted by the ACCORD trial [3]. In fact, one retrospective Canadian review demonstrated that the febrile neutropenia rate was approximately double that seen in the trial [10]. Reasons for the discrepancy may relate to less stringent patient selection in a real-world setting. A recent retrospective analysis suggested that an RDI > 80% predicted frequent febrile neutropenia and noted that primary prophylaxis with GCSF could be a consideration in this situation [24].

Subsequent studies have been conducted showing that modified FOLFIRINOX in the metastatic setting has an improved safety profile with maintained efficacy, even in the absence of prophylactic GCSF [23]. Many cancer centers have adopted this modified regimen as a standard of care for both unresectable/metastatic and adjuvant pancreatic cancer more recently. The authors of this paper are in support of this practice. Interestingly, in the 2020 ASCO guidelines [25], there is no mention of upfront dose-adjusted FFX or modified FFX for patients with metastatic cancer and good ECOG PS 0–1, though it does comment that for patients with ECOG PS 2, consideration should be made for first-line gemcitabine. Further, there is a recommendation in this setting that if first-line gemcitabine plus a second agent is entertained (such as nab-paclitaxel, capecitabine, or erlotinib), then proactive dose and schedule adjustments should be considered to minimize toxicity. It is clear that upfront adjustments for toxicity minimization are in principle acceptable.

Limitations of this study include its retrospective nature, meaning causal associations cannot be inferred. Due to the retrospective nature of the analysis, reasons for dose adjustments and discontinuation were often not available in the medical records, which limited the analysis of characteristics leading to treatment adjustments. Similarly, while our cohort included patients from eight cancer centers in four Canadian provinces, it may not be representative of the entire Canadian landscape, as some provinces were not captured in the database.

## 5. Conclusions

In summary, the main first-line systemic therapy treatments for advanced pancreas cancer include FOLFIRINOX or Gemcitabine plus nab-Paclitaxel. Despite advances, outcomes remain sub-optimal. Medical oncologists in Canada historically prescribed FFX to patients with advanced pancreatic cancer in a fashion that was not uniform, prior to the emergence of any evidence for upfront dose reductions. Some patients were treated with initial standard dosing and the remainder were treated with upfront dose alterations which were variable, reflecting differing cultures across cancer centers, different physician preferences, and different patient characteristics. In the years that followed, some centers, including our own, have started routinely using modified FOLFIRINOX in the metastatic setting for tolerance. A clinical practice guideline addressing dosing strategies would help guide clinicians on the optimal method of initiating and managing FFX and other regimens for patients with advanced pancreatic cancer. Availability of molecular testing and opportunities for clinical trials should also be pursued in this population. Similarly, further studies identifying patients most likely to tolerate and benefit from standard- vs. adjusted-dose FFX and other treatments are needed to personalize pancreas cancer management [31,32,33].

## Figures and Tables

**Table 1:** Baseline characteristics.

Characteristic	Category	Results
		(N = 224)
Age at FFX, years	median (range)	61 (24−80)
	≥65, n (%)	78 (35%)
	≥75, n (%)	15 (7%)
Female, n (%)		95 (43%)
BMI, kg/m^2^	median (range)	24 (15−63)
ECOG PS, n (%)	0	129 (58%)
	1	84 (38%)
	2+	10 (4%)
Stage of disease, n (%)	LAPC	47 (21%)
	mPC	176 (79%)
Similarity with ACCORD trial *	ACCORD positive	94 (53%)
	ACCORD negative	82 (47%)
T stage at FFX start, n (%)	T1	10 (5%)
	T2	27 (12%)
	T3	81 (36%)
	T4	83 (37%)
	TX	22 (10%)
N stage at FFX start, n (%)	N0	77 (35%)
	N1	117 (52%)
	NX	29 (13%)
Primary tumor site within pancreas, n (%)	Head	130 (58%)
	Body/Tail	93 (42%)
No. of metastatic sites *, n (%)	1	66 (38%)
	2	22 (12%)
	≥3	88 (50%)
Liver-only metastases *, n (%)		82 (47%)
Curative surgery, n (%)		43 (19%)
Adjuvant chemotherapy, n (%)	5-Fluorouracil	6 (2%)
	Gemcitabine	31 (14%)
Biliary stent, n (%)		66 (30%)

* In metastatic pancreas cancer only. FFX, FOLFIRINOX; LAPC, locally advanced pancreatic cancer; mPC, metastatic pancreatic cancer; ACCORD positive, fulfills ACCORD trial inclusion criteria; ACCORD negative, does not fulfill ACCORD trial inclusion criteria.

**Table 2:** Baseline characteristics by ACCORD inclusion criteria and FFX starting dose.

Characteristic	Category	FFX Starting Dose			ACCORD Inclusion Criteria		
		Standard (n = 69)	Reduced (n = 62)	w/o 5FUb (n = 92)	ACCORD+ (n = 94)	ACCORD− (n = 82)	LAPC
							(n = 47)
Age at FFX, years	median (range)	60 (34−77)	60 (39−79)	62 (24−80)	59 (34−75)	61 (24−80)	64 (41−79)
	≥65, n (%)	22 (32%)	22 (35%)	34 (37%)	28 (30%)	29 (35%)	21 (45%)
	≥75, n (%)	3 (4%)	5 (8%)	7 (8%)	---	11 (13%)	4 (9%)
Female, n (%)		21 (30%)	32 (52%)	42 (46%)	43 (46%)	34 (41%)	18 (38%)
BMI, kg/m^2^	median (range)	25 (16−40)	24 (15−63)	23 (17−39)	25 (16−40)	24 (15−39)	23 (17−63)
ECOG PS	0	41 (59%)	27 (44%)	61 (66%)	53 (56%)	43 (52%)	33 (70%)
	1	27 (39%)	32 (52%)	25 (27%)	41 (44%)	30 (37%)	13 (28%)
	2+	1 (1%)	3 (5%)	5 (5%)	---	9 (11%)	1 (2%)
Stage of disease, n (%)	LAPC	14 (20%)	7 (11%)	26 (28%)	---	---	47 (21%)
	mPC	55 (80%)	55 (89%)	66 (72%)	94 (100%)	82 (100%)	---
Similarity with ACCORD trial *	ACCORD positive	39 (71%)	21 (38%)	34 (52%)			
	ACCORD negative	16 (29%)	34 (62%)	32 (48%)			
T stage at FFX start, n (%)	T1	2 (3%)	4 (6%)	4 (4%)	9 (9%)	0 (0%)	1 (2%)
	T2	7 (10%)	12 (19%)	8 (9%)	12 (13%)	11 (13%)	4 (9%)
	T3	16 (23%)	19 (31%)	46 (50%)	33 (35%)	30 (37%)	18 (38%)
	T4	36 (52%)	25 (40%)	22 (34%)	29 (31%)	31 (38%)	23 (49%)
	TX	8 (12%)	2 (3%)	12 (13%)	11 (12%)	10 (12%)	1 (2%)
N stage at FFX start, n (%)	N0	22 (32%)	26 (41%)	29 (32%)	31 (33%)	28 (34%)	18 (38%)
	N1	39 (57%)	29 (47%)	49 (53%)	51 (54%)	40 (49%)	26 (55%)
	NX	8 (12%)	7 (11%)	14 (15%)	12 (13%)	14 (17%)	3 (7%)
Primary tumor site, n (%)	Head	41 (59%)	32 (52%)	57 (62%)	48 (51%)	48 (59%)	34 (72%)
	Body/Tail	28 (41%)	30 (48%)	35 (38%)	46 (49%)	34 (41%)	13 (28%)
No. of metastatic sites *, n (%)	1	30 (55%)	24 (44%)	12 (18%)	39 (42%)	27 (33%)	---
	2	10 (18%)	5 (9%)	7 (11%)	8 (8%)	14 (17%)	---
	≥3	15 (27%)	26 (47%)	47 (71%)	47 (50%)	35 (43%)	---
Liver-only metastases *, n (%)		27 (49%)	28 (51%)	27 (41%)	47 (50%)	35 (43%)	---
Curative surgery, n (%)		14 (20%)	10 (16%)	19 (21%)	19 (20%)	13 (16%)	11 (23%)
Adjuvant chemotherapy, n (%)	5-Fluorouracil	4 (6%)	0 (0%)	2 (2%)	2 (2%)	1 (1%)	3 (6%)
	Gemcitabine	9 (13%)	9 (15%)	13 (14%)	15 (16%)	8 (10%)	8 (17%)
Biliary stent, n (%)		16 (23%)	21 (34%)	29 (32%)	20 (21%)	30 (37%)	16 (34%)

* In metastatic pancreas cancer only. Abbreviations: w/o, without; 5-FU, 5-fluorouracil; ECOG PS, Eastern Cooperative Oncology Group Performance Status.

**Table 3:** FFX administration.

Administration	Categories	Cohort
		(N = 224)
FFX starting dose (categorized), n (%)	Standard	69 (31%)
	Reduced	62 (28%)
	Standard w/o 5-FU bolus	20 (9%)
	Reduced w/o 5-FU bolus	72 (32%)
FFX starting dose *, n (%)	90–100%	73 (33%)
	70–90%	71 (32%)
	Less than 70%	79 (35%)
FFX duration, cycles	median (range)	7 (1–48)
FFX duration, months	median (range)	8.4 (0.5–35.9)
1st dose reduction, cycles	median (range)	8 (1–32)
1st dose delay, cycles	median (range)	5 (1–22)
GCSF prophylaxis, n (%)	Primary	54 (24%)
	Secondary	37 (17%)

* Average starting dose intensity for whole regimen where each component (5-FU bolus, 5-FU infusion, Irinotecan, Oxaliplatin) was weighted equally. Abbreviations: FFX, FOLFIRINOX; w/o, without; 5-FU, 5-fluorouracil; GCSF, granulocyte colony stimulating factor.

**Table 4:** Relative dose intensity.

Drug(s)	Cycle 1 (N = 223)	Cycle 3 (N = 124)	Cycle 6 (N = 68)
FFX	71%	64%	59%
Oxaliplatin	88%	80%	77%
Irinotecan	80%	75%	72%
5-FU Bolus	61%	29%	12%
5-FU Infusion	92%	80%	85%

Abbreviations: FFX, FOLFIRINOX; 5-FU, 5-fluorouracil.

## Data Availability

Data is not available due to privacy restrictions.

## References

[1] Nuttall R., Bryan S., Dale D., De P., Demers A., Ellison L. (2017). Canadian Cancer Society’s Advisory Committee on Cancer Statistics. Canadian Cancer Statistics 2017.

[2] Gnanamony M., Gondi C.S. (2017). Chemoresistance in pancreatic cancer: Emerging concepts. Oncol. Lett..

[3] Conroy T., Desseigne F., Ychou M., Bouche O., Guimbaud R., Becouarn Y., Adenis A., Raoul J.L., Gourgou-Bourgade S., de la Fouchardiere C. (2011). FOLFIRINOX versus gemcitabine for metastatic pancreatic cancer. N. Engl. J. Med..

[4] Von Hoff D.D., Ervin T., Arena F.P., Chiorean E.G., Infante J., Moore M., Seay T., Tjulandin S.A., Ma W.W., Saleh M.N. (2013). Increased survival in pancreatic cancer with nab-paclitaxel plus gemcitabine. N. Engl. J. Med..

[5] Sohal D.P.S., Kennedy E.B., Cinar P., Conroy T., Copur M.S., Crane C.H., Garrido-Laguna I., Lau M.W., Johnson T., Krishnamurthi S. (2020). Metastatic Pancreatic Cancer: ASCO Guideline Update. J. Clin. Oncol..

[6] Burris H.A., Moore M.J., Andersen J., Green M.R., Rothenberg M.L., Modiano M.R., Cripps M.C., Portenoy R.K., Storniolo A.M., Tarassoff P. (1997). Improvements in survival and clinical benefit with gemcitabine as first-line therapy for patients with advanced pancreas cancer: A randomized trial. J. Clin. Oncol..

[7] Suker M., Beumer B.R., Sadot E., Marthey L., Faris J.E., Mellon E.A., El-Rayes B.F., Wang-Gillam A., Lacy J., Hosein P.J. (2016). FOLFIRINOX for locally advanced pancreatic cancer: A systematic review and patient-level meta-analysis. Lancet Oncol..

[8] Marthey L., Sa-Cunha A., Blanc J.F., Gauthier M., Cueff A., Francois E., Trouilloud I., Malka D., Bachet J.B., Coriat R. (2015). FOLFIRINOX for locally advanced pancreatic adenocarcinoma: Results of an AGEO multicenter prospective observational cohort. Ann. Surg. Oncol..

[9] Balaban E.P., Mangu P.B., Khorana A.A., Shah M.A., Mukherjee S., Crane C.H., Javle M.M., Eads J.R., Allen P., Ko A.H. (2016). Locally Advanced, Unresectable Pancreatic Cancer: American Society of Clinical Oncology Clinical Practice Guideline. J. Clin. Oncol..

[10] Amireault C., Beaudet J., Gaudet G., Raymond N., Ayoub J.-P., Letourneau R., Loungnarath R., Tehfe M., Aubin F. (2014). FOLFIRINOX in the real-world setting: The multicentric experience of six Canadian institutions. ASCO Meet. Abstr..

[11] Marsh Rde W., Talamonti M.S., Katz M.H., Herman J.M. (2015). Pancreatic cancer and FOLFIRINOX: A new era and new questions. Cancer Med..

[12] Hryniuk W., Levine M.N. (1986). Analysis of dose intensity for adjuvant chemotherapy trials in stage II breast cancer. J. Clin. Oncol..

[13] Hryniuk W.M. (1987). Average relative dose intensity and the impact on design of clinical trials. Semin. Oncol..

[14] Anuradha S., Donovan P.J., Webb P.M., Brand A.H., Goh J., Friedlander M., Oehler M.K., Quinn M., Steer C., Jordan S.J. (2016). Variations in adjuvant chemotherapy and survival in women with epithelial ovarian cancer—A population-based study. Acta Oncol..

[15] Fauci J.M., Whitworth J.M., Schneider K.E., Subramaniam A., Zhang B., Frederick P.J., Kilgore L.C., Straughn J.M. (2011). Prognostic significance of the relative dose intensity of chemotherapy in primary treatment of epithelial ovarian cancer. Gynecol. Oncol..

[16] Lepage E., Gisselbrecht C., Haioun C., Sebban C., Tilly H., Bosly A., Morel P., Herbrecht R., Reyes F., Coiffier B. (1993). Prognostic significance of received relative dose intensity in non-Hodgkin’s lymphoma patients: Application to LNH-87 protocol. The GELA. (Groupe d’Etude des Lymphomes de l’Adulte). Ann. Oncol..

[17] Aspinall S.L., Good C.B., Zhao X., Cunningham F.E., Heron B.B., Geraci M., Passero V., Stone R.A., Smith K.J., Rogers R. (2015). Adjuvant chemotherapy for stage III colon cancer: Relative dose intensity and survival among veterans. BMC Cancer.

[18] Havrilesky L.J., Reiner M., Morrow P.K., Watson H., Crawford J. (2015). A review of relative dose intensity and survival in patients with metastatic solid tumors. Crit. Rev. Oncol. Hematol..

[19] Schemper M., Smith T.L. (1996). A note on quantifying follow-up in studies of failure time. Control Clin. Trials.

[20] Karim S., Zhang-Salomans J., Biagi J.J., Asmis T., Booth C.M. (2018). Uptake and Effectiveness of FOLFIRINOX for Advanced Pancreatic Cancer: A Population-based Study. Clin. Oncol. (R. Coll. Radiol.).

[21] Al-Baimani K., Jonker H., Zhang T., Goss G.D., Laurie S.A., Nicholas G., Wheatley-Price P. (2018). Are clinical trial eligibility criteria an accurate reflection of a real-world population of advanced non-small-cell lung cancer patients?. Curr. Oncol..

[22] Okusaka T., Ikeda M., Fukutomi A., Ioka T., Furuse J., Ohkawa S., Isayama H., Boku N. (2014). Phase II study of FOLFIRINOX for chemotherapy-naive Japanese patients with metastatic pancreatic cancer. Cancer Sci..

[23] Ozaka M., Ishii H., Sato T., Ueno M., Ikeda M., Uesugi K., Sata N., Miyashita K., Mizuno N., Tsuji K. (2018). A phase II study of modified FOLFIRINOX for chemotherapy-naive patients with metastatic pancreatic cancer. Cancer Chemother. Pharmacol..

[24] Lee J.C., Kim J.W., Ahn S., Kim H.W., Lee J., Kim Y.H., Paik K.H., Kim J., Hwang J.H. (2017). Optimal dose reduction of FOLFIRINOX for preserving tumour response in advanced pancreatic cancer: Using cumulative relative dose intensity. Eur. J. Cancer.

[25] Sohal D.P., Mangu P.B., Khorana A.A., Shah M.A., Philip P.A., O’Reilly E.M., Uronis H.E., Ramanathan R.K., Crane C.H., Engebretson A. (2016). Metastatic Pancreatic Cancer: American Society of Clinical Oncology Clinical Practice Guideline. J. Clin. Oncol..

[26] Conroy T., Hammel P., Hebbar M., Ben Abdelghani M., Wei A.C., Raoul J.L., Chone L., Francois E., Artru P., Biagi J.J. (2018). FOLFIRINOX or Gemcitabine as Adjuvant Therapy for Pancreatic Cancer. N. Engl. J. Med..

[27] Kang H., Jo J.H., Lee H.S., Chung M.J., Bang S., Park S.W., Song S.Y., Park J.Y. (2018). Comparison of efficacy and safety between standard-dose and modified-dose FOLFIRINOX as a first-line treatment of pancreatic cancer. World J. Gastrointest. Oncol..

[28] Uson Junior P.L.S., Rother E.T., Maluf F.C., Bugano D.D.G. (2018). Meta-analysis of Modified FOLFIRINOX Regimens for Patients With Metastatic Pancreatic Cancer. Clin. Color. Cancer.

[29] Ulusakarya A., Teyar N., Karaboue A., Haydar M., Krimi S., Biondani P., Gumus Y., Almohamad W., Morere J.F. (2018). Patient-tailored FOLFIRINOX in the first-line treatment of patients (pts) with advanced pancreatic cancer (PC). J. Clin. Oncol..

[30] Smith T.J., Bohlke K., Lyman G.H., Carson K.R., Crawford J., Cross S.J., Goldberg J.M., Khatcheressian J.L., Leighl N.B., Perkins C.L. (2015). Recommendations for the Use of WBC Growth Factors: American Society of Clinical Oncology Clinical Practice Guideline Update. J. Clin. Oncol..

[31] Gu A., Li J., Qiu S., Hao S., Yue Z.Y., Zhai S., Li M.Y., Liu Y. (2024). Pancreatic cancer environment: From patient-derived models to single-cell omics. Mol. Omics.

[32] Chen M., Sun Y., Liu H. (2023). Cell membrane biomimetic nanomedicines for cancer phototherapy. Interdiscip. Med..

[33] Shubhra Q.T.H., Cai X., Cai Q. (2024). Next-Generation Tumor Targeting with Genetically Engineered Cell Membrane-Coated Nanoparticles. Biodes Res..

